# Breast cancer risk genes affecting individual radiosensitivity

**DOI:** 10.1038/s41598-026-57275-x

**Published:** 2026-06-14

**Authors:** Ramona K. G. Vogel, Niklas Amann, Tara Zuhair Kassem, Paul Gaß, Theresa Mayo, Felix Woltereck, Carolin C. Hack, Rainer Fietkau, Stefanie Corradini, Luitpold Distel, Lukas C. F. Kuhlmann

**Affiliations:** 1 Department of Radiation Oncology, Universitätsklinikum Erlangen, Friedrich-Alexander-Universität Erlangen-Nürnberg (FAU), Universitätsstraße 27, D-91054 Erlangen, Germany; 2 Department of Obstetrics and Gynecology, Comprehensive Cancer Center Erlangen-EMN, Universitätsklinikum Erlangen, Friedrich-Alexander-Universität Erlangen-Nürnberg (FAU), Universitätsstraße 21/23, D-91054 Erlangen, Germany; 3 Klinikum Chemnitz gGmbH, Medizincampus Chemnitz der Technische Universität Dresden, Flemmingstraße 2, D-09116 Chemnitz, Germany; 4 Department of Obstetrics and Gynecology, Klinikum Rechts der Isar der Technischen Universität München (TUM), Ismaninger Str. 22, D-81675 Munich, Germany

**Keywords:** Breast cancer risk genes, Radiosensitivity, *BRCA1*, *BRCA2*, Fluorescence in situ hybridization, Chromosomal aberrations, Cancer, Genetics, Oncology

## Abstract

Increased radiosensitivity can cause adverse radiotherapy effects. Heterozygote variants in breast cancer risk genes increase cancer risk and may affect radiosensitivity. This study assessed their impact on individual radiosensitivity to determine the radiation risk for gene carriers. Radiosensitivity was analyzed in 273 patients with breast cancer risk gene variants. Using fluorescence in situ hybridization (FiSH), chromosomal aberrations were quantified as breaks per metaphase (B/M) after ex vivo irradiation of blood lymphocytes. Results were compared with healthy controls and breast cancer cases, both without confirmed non-carrier status, limiting gene-specific conclusions. Gene carriers showed slightly increased radiosensitivity (mean 0.488 B/M ± 0.134) compared with healthy controls (0.411 B/M ± 0.088; *p* < 0.0001) but similar to breast cancer control group (0.498 B/M ± 0.192; *p* = 0.563). In *BRCA1/2* carriers, radiosensitivity was slightly increased, while single cases in *BARD1*, *RAD51*, and *MSH *variants suggested a possible increase (*p* < 0.003). Radiosensitivity was influenced by gene locus, variant type, age, and cancer history. 24.5% of carriers exceeded a cutoff of ≥ 0.55 B/M, where dose reduction could be considered. Radiosensitivity of breast cancer risk gene carriers varies and is slightly increased. Individuals with increased radiosensitivity risk should consider testing.

## Introduction

There are many known breast cancer risk genes, some with a very high risk of developing cancer at a young age. The lifetime risk for breast cancer is approximately 12% in the general population and rises in carriers of pathogenetic variants. An increased risk has been confirmed for 12 genes: *ATM*,* BARD1*,* BRCA1*,* BRCA2*,* CHEK2*,* MSH6*,* NF1*,* PALB2*,* PTEN*,* RAD51C*,* RAD51D* and *TP53*^1^. In addition, an association with *CDH1* for breast cancer was found in the US population^2^. Around 5–10% of breast cancers are hereditary, with 3–4% caused by pathogenic variants in the *BRCA1* and *BRCA2* genes^3^. One in 60 people in the general population carries a risk gene^2^. Estimates for *BRCA1* variant carriers range from 0.07% to 0.09%, with 0.14% to 0.22% for *BRCA2* carriers^4^. *BRCA1*,* BRCA2*, *PALB2* and *TP53* are high-risk genes, with a risk of over 30% of breast cancer by the age of 80. The estimated lifetime risks for *BRCA1* and *BRCA2* were 55% and 45%, respectively. For *ATM*,* BARD1*,* CHEK2*,* RAD51C* and *RAD51D* the absolute risk at age 80 years was estimated to be in a moderate range between 17% and 30%. *BRCA1* confers a high risk, often before the age of 40, as well as *BRCA2* and *PALB2*, but usually a little later than *BRCA1*. *TP53*, which causes Li-Fraumeni syndrome, is associated with very early childhood cancers. Apart from increasing breast cancer risk, these variants can cause other cancers. For example, *BRCA1* and *BRCA2* increase the risk of ovarian cancer and some *CHEK2* variants increase the risk of colorectal cancer, while *ATM* variants may be linked to pancreatic cancer^1,5^.

Women who carry breast cancer genes have a higher risk of developing breast cancer and therefore requiring radiation therapy. Approximately 70% of breast cancer patients undergo treatment with adjuvant radiation^6^. Breast cancer risk genes are involved in DNA repair and cell cycle control. Therefore, variants in these genes lead to difficulties in processing DNA damage, followed by an accumulation of mis repaired DNA damage and the development of cancer over time^7^. Treatment for breast cancer includes chemotherapeutics, surgery and radiotherapy. Radiotherapy is the first-line treatment for breast cancer and is used to control tumors locally. This allows breast conservation surgery instead of mastectomy and it reduces the risk of recurrence to almost the same level. The repair mechanism in breast cancer cells is less effective than in healthy cells, causing cell death and making radiotherapy very effective^8^.

Genetic variants influence radiosensitivity and have an impact on both the effectiveness of tumor treatment and the risk of undesired side effects. Studies indicate that variants in DNA repair genes such as *ATM* and *BRCA1/2* can make solid tumors more sensitive to radiation^9^. These results suggest that tumors’ response to radiotherapy may be influenced by these variants, which should be considered in treatment planning. In addition, undesired side effects are often observed in patients with variants in DNA repair genes^10^. Increased radiosensitivity results in a higher risk of side effects and these patients should be identified for dose adaption and better monitoring before starting therapy^11^. It has already been shown that variants in the *BRCA1* and *BRCA2* genes confer a slightly increased level of radiosensitivity^12^. Therefore, we wanted to increase the size of the cohort in *BRCA* patients to confirm these previous findings and to expand the study to other breast cancer risk genes. The aim of this study was to determine whether specific breast cancer risk genes involved in DNA repair mechanisms cause increased radiosensitivity compared to healthy individuals or breast cancer patients with unknown variant status. We are also interested in whether a specific variant site in the genes may be associated with increased radiosensitivity.

## Materials and methods

### Patient recruitment

The cohort contains radiosensitivity data from 273 patients (mean age 47 years, range 10–91 years) with breast cancer risk genes, studied by blood samples in the period from 2016 to 2025. The study included 267 women and six men. Of these, 170 patients had an oncological disease (150 patients with breast cancer and 20 with other cancers, including ovarian, uterine, fallopian tube, prostate, renal, thyroid, rectal and gastric cancers, as well as medulloblastoma) and 103 had no known oncological disease. Most breast cancer patients had early-stage disease in Tis, T1 or T2 (62%) and no evidence of tumor progression. In total, 198 samples came from genetic counselling at the Department of Obstetrics and Gynecology of the Universitätsklinikum Erlangen and 16 samples were sent by the TUM Klinikum Rechts der Isar in Munich. 59 patients were collected in our radiotherapy department or referred from other clinics for radiosensitivity testing. The results contain previously published radiosensitivity data from 64 patients with *BRCA1* and *BRCA2* variants^12^ (Open diamond symbol indicates this data). The entire cohort includes the following gene variants: *ATM* heterozygotes (*ATM*_*het*_), *BARD1*,* BRIP1*,* BRCA1/2*,* CHEK2*,* CHD1*,* MSH*,* NF1*,* PALB2*,* PMS2*,* RAD51C/D* and *TP53* according to the criteria of the American College of Medical Genetics and Genomics. Genetic testing was generally performed either because of an oncologic disease or because of a known risk situation. Where available, clinical data were extracted from electronic health records and hospital databases (Table 1). Patients gave written informed consent to participate in the study. Consent was obtained from the minors by both the minors themselves and their parents or guardians. The study was approved by the Ethics Committee of the Friedrich-Alexander-Universität Erlangen-Nürnberg (FAU) (21_19 B; 18.02.2019). Existing results from 211 healthy controls (mean age: 50.3 years) and 147 breast cancer patients (mean age: 57.3 years) were used for comparison. They were previously published and collected at the Department of Radiation Oncology, Universitätsklinikum Erlangen^13,14^. In both comparison groups, a possible variant status is unknown. The prevalence of variants is expected to be low in the healthy control group and higher in the breast cancer group. The comparison groups were chosen because they allow to draw conclusions about the influence of the variant status as well as the presence of an oncological disease^12^.

**Table 1:** Patients’ characteristics of the breast cancer risk gene carriers. het = heterozygote; B/M = Breaks per metaphase; Tis = Tumor in situ; SD = standard deviation.

variable		All risk genes (%)	*ATM*_*het*_ (%)	*BARD1* (%)	*BRCA1* (%)	*BRCA2* (%)	*BRCA* undefined (%)	*BRIP1* (%)	*CHD1* (%)	*CHEK2* (%)	*MSH* (%)	*NF1* (%)	*PALB2* (%)	*PMS2* (%)	*RAD51C* (%)	*RAD51D* (%)	*TP53* (%)	Double heterozygote (%)
n =		273	40	3	87	61	5	6	2	23	16	1	9	1	6	3	3	7
Sex	Men	6 (2.2)	0 (0)	0 (0)	0 (0)	0 (0)	1 (20)	0 (0)	0 (0)	0 (0)	4 (25)	0 (0)	0 (0)	1 (100)	0 (0)	0 (0)	0 (0)	0 (0)
	Female	267 (97.8)	40 (100)	3 (100)	87 (100)	61 (100)	4 (80)	6 (100)	2 (100)	23 (100)	12 (75)	1 (100)	9 (100)	0 (0)	6 (100)	3 (100)	3 (100)	7 (100)
Age	Mean age (years)	47	50	52	45	47	59	47	41	47	50	34	42	72	47	38	43	44
	Range (years)	10–91	32–73	44–59	19–91	24–78	47–77	27–59	35–46	27–68	10–75	-	21–62	-	32–63	30–43	36–53	32–62
Menopause status	Premenopausal	162 (59.3)	26 (65)	2 (66.7)	50 (57.5)	33 (54.1)	2 (40)	3 (50)	2 (100)	15 (65.2)	5 (31.3)	1 (100)	6 (66.7)	0 (0)	5 (83.3)	3 (100)	3 (100)	6 (85.7)
	Postmenopausal	103 (37.7)	12 (30)	1 (33.3)	37 (42.5)	28 (45.9)	2 (40)	3 (50)	0 (0)	8 (34.8)	7 (43.8)	0 (0)	3 (33.3)	0 (0)	1 (16.7)	0 (0)	0 (0)	1 (14.3)
	Not known	2 (0.7)	2 (5)	0 (0)	0 (0)	0 (0)	0 (0)	0 (0)	0 (0)	0 (0)	0 (0)	0 (0)	0 (0)	0 (0)	0 (0)	0 (0)	0 (0)	0 (0)
Oncology	Oncological	170 (62.3)	32 (80)	1 (33.3)	52 (59.8)	34 (55.7)	5 (100)	4 (66.7)	1 (50)	14 (60.9)	8 (50)	0 (0)	6 (66.7)	1 (100)	5 (83.3)	1 (33.3)	2 (66.7)	4 (57.1)
	Including breast cancer	150 (54.9)	32 (80)	1 (33.3)	44 (50.6)	32 (52.5)	4 (80)	3 (50)	1 (50)	13 (56.5)	3 (18.8)	0 (0)	6 (66.7)	1 (100)	4 (66.7)	1 (33.3)	1 (33.3)	4 (57.1)
	No oncological disease known	103 (37.7)	8 (20)	2 (66.7)	35 (40.2)	27 (44.3)	0 (0)	2 (33.3)	1 (50)	9 (39.1)	8 (50)	1 (100)	3 (33.3)	0 (0)	1 (16.7)	2 (66.7)	1 (33.3)	3 (42.9)
	Multiple oncological diseases	41 (15)	5 (12.5)	0 (0)	17 (19.5)	9 (14.8)	0 (0)	0 (0)	0 (0)	3 (13)	2 (12.5)	0 (0)	3 (33.3)	0 (0)	0 (0)	0 (0)	1 (33.3)	1 (14.3)
TNM status of breast cancer patients	Tis	7 (2.6)	2 (5)	0 (0)	3 (3.4)	1 (1.6)	0 (0)	0 (0)	0 (0)	0 (0)	0 (0)	0 (0)	0 (0)	0 (0)	0 (0)	0 (0)	1 (33.3)	0 (0)
	T1	44 (16.1)	6 (15)	1 (33.3)	11 (12.6)	13 (21.3)	0 (0)	1 (16.7)	0 (0)	6 (26.1)	0 (0)	0 (0)	3 (33.3)	0 (0)	0 (0)	0 (0)	0 (0)	3 (42.9)
	T2	42 (15.4)	2 (5)	0 (0)	23 (26.4)	8 (13.1)	0 (0)	1 (16.7)	0 (0)	1 (4.3)	1 (6.3)	0 (0)	2 (22.2)	1 (100)	2 (33.3)	1 (33.3)	0 (0)	0 (0)
	T3	4 (1.5)	1 (2.5)	0 (0)	2 (2.3)	0 (0)	0 (0)	0 (0)	0 (0)	1 (4.3)	0 (0)	0 (0)	0 (0)	0 (0)	0 (0)	0 (0)	0 (0)	0 (0)
	T4	0 (0)	0 (0)	0 (0)	0 (0)	0 (0)	0 (0)	0 (0)	0 (0)	0 (0)	0 (0)	0 (0)	0 (0)	0 (0)	0 (0)	0 (0)	0 (0)	0 (0)
	Tx	53 (19.4)	21 (52.5)	0 (0)	5 (5.7)	10 (16.4)	4 (80)	1 (16.7)	1 (50)	5 (21.7)	2 (12.5)	0 (0)	1 (11.1)	0 (0)	2 (33.3)	0 (0)	0 (0)	1 (14.3)
	N0	69 (25.3)	6 (15)	1 (33.3)	28 (32.2)	16 (26.2)	0 (0)	2 (33.3)	0 (0)	6 (26.1)	0 (0)	0 (0)	3 (33.3)	1 (100)	2 (33.3)	0 (0)	1 (33.3)	3 (42.9)
	N+	27 (9.9)	4 (10)	0 (0)	11 (12.6)	6 (9.8)	0 (0)	0 (0)	0 (0)	2 (8.7)	1 (6.3)	0 (0)	2 (22.2)	0 (0)	0 (0)	1 (33.3)	0 (0)	0 (0)
	Nx	54 (19.8)	22 (55)	0 (0)	5 (5.7)	10 (16.4)	4 (80)	1 (16.7)	1 (50)	5 (21.7)	2 (12.5)	0 (0)	1 (11.1)	0 (0)	2 (33.3)	0 (0)	0 (0)	1 (14.3)
	M0	73 (26.7)	9 (22.5)	1 (33.3)	32 (36.8)	18 (29.5)	0 (0)	2 (33.3)	0 (0)	5 (21.7)	1 (6.3)	0 (0)	2 (22.2)	0 (0)	1 (16.7)	0 (0)	1 (33.3)	1 (14.3)
	M+	7 (2.6)	2 (5)	0 (0)	1 (1.1)	1 (1.6)	0 (0)	0 (0)	0 (0)	1 (4.3)	0 (0)	0 (0)	0 (0)	0 (0)	0 (0)	1 (33.3)	0 (0)	1 (14.3)
	Mx	70 (25.6)	21 (52.5)	0 (0)	11 (12.6)	13 (21.3)	4 (80)	1 (16.7)	1 (50)	7 (30.4)	2 (12.5)	0 (0)	4 (44.4)	1 (100)	3 (50)	0 (0)	0 (0)	2 (28.6)
Receptors	ER or PR positive	50 (18.3)	8 (20)	0 (0)	9 (10.3)	14 (23)	0 (0)	2 (33.3)	0 (0)	6 (26.1)	1 (6.3)	0 (0)	3 (33.3)	1 (100)	1 (16.7)	1 (33.3)	0 (0)	4 (57.1)
	HER2/neu positive	12 (4.4)	2 (5)	0 (0)	6 (6.9)	3 (4.9)	0 (0)	0 (0)	0 (0)	1 (4.3)	0 (0)	0 (0)	0 (0)	0 (0)	0 (0)	0 (0)	0 (0)	0 (0)
	Not known	53 (19.4)	23 (57.5)	0 (0)	6 (6.9)	9 (14.8)	3 (60)	1 (16.7)	1 (50)	5 (21.7)	2 (12.5)	0 (0)	0 (0)	0 (0)	2 (33.3)	0 (0)	1 (33.3)	0 (0)
Molecular subtype	Luminal A	12 (4.4)	2 (5)	0 (0)	0 (0)	3 (4.9)	0 (0)	2 (33.3)	0 (0)	3 (13)	0 (0)	0 (0)	0 (0)	1 (100)	0 (0)	0 (0)	0 (0)	1 (14.3)
	Luminal B (HER2-)	29 (10.6)	4 (10)	0 (0)	7 (8)	8 (13.1)	0 (0)	0 (0)	0 (0)	2 (8.7)	1 (6.3)	0 (0)	3 (33.3)	0 (0)	1 (16.7)	1 (33.3)	0 (0)	2 (28.6)
	Luminal B (HER2+)	7 (2.6)	2 (5)	0 (0)	2 (2.3)	2 (3.3)	0 (0)	0 (0)	0 (0)	1 (4.3)	0 (0)	0 (0)	0 (0)	0 (0)	0 (0)	0 (0)	0 (0)	0 (0)
	HER2-positiv (non-luminal)	5 (1.8)	0 (0)	0 (0)	4 (4.6)	1 (1.6)	0 (0)	0 (0)	0 (0)	0 (0)	0 (0)	0 (0)	0 (0)	0 (0)	0 (0)	0 (0)	0 (0)	0 (0)
	Triple-negative	41 (15)	1 (2.5)	1 (33.3)	24 (27.6)	8 (13.1)	1 (20)	0 (0)	0 (0)	2 (8.7)	0 (0)	0 (0)	3 (33.3)	0 (0)	1 (16.7)	0 (0)	0 (0)	0 (0)
	Not known	56 (20.5)	23 (57.5)	0 (0)	7 (8)	10 (16.4)	3 (60)	1 (16.7)	1 (50)	5 (21.7)	2 (12.5)	0 (0)	0 (0)	0 (0)	2 (33.3)	0 (0)	1 (33.3)	1 (14.3)
Grading	G1	6 (2.2)	1 (2.5)	0 (0)	1 (1.1)	2 (3.3)	0 (0)	0 (0)	0 (0)	2 (8.7)	0 (0)	0 (0)	0 (0)	0 (0)	0 (0)	0 (0)	0 (0)	0 (0)
	G2	26 (9.5)	4 (10)	0 (0)	5 (5.7)	4 (6.6)	0 (0)	2 (33.3)	0 (0)	4 (17.4)	1 (6.3)	0 (0)	3 (33.3)	1 (100)	0 (0)	0 (0)	0 (0)	2 (28.6)
	G3	60 (22)	5 (12.5)	1 (33.3)	30 (34.5)	15 (24.6)	0 (0)	0 (0)	0 (0)	2 (8.7)	0 (0)	0 (0)	3 (33.3)	0 (0)	1 (16.7)	1 (33.3)	1 (33.3)	1 (14.3)
	Not known	58 (21.2)	22 (55)	0 (0)	8 (9.2)	11 (18)	4 (80)	1 (16.7)	1 (50)	5 (21.7)	2 (12.5)	0 (0)	0 (0)	0 (0)	3 (50)	0 (0)	0 (0)	1 (14.3)
Ki67-Index	Ki67-Index (> 20%)	77 (28.2)	7 (17.5)	1 (33.3)	34 (39.1)	17 (27.9)	1 (20)	0 (0)	0 (0)	5 (21.7)	1 (6.3)	0 (0)	6 (66.7)	0 (0)	2 (33.3)	1 (33.3)	0 (0)	2 (28.6)
	Ki67-Index (< 20%)	15 (5.5)	2 (5)	0 (0)	1 (1.1)	5 (8.2)	0 (0)	2 (33.3)	0 (0)	3 (13)	0 (0)	0 (0)	0 (0)	1 (100)	0 (0)	0 (0)	0 (0)	1 (14.3)
	Ki67-Index not known	58 (21.2)	23 (57.5)	0 (0)	10 (11.5)	9 (14.8)	3 (60)	1 (16.7)	1 (50)	5 (21.7)	2 (12.5)	0 (0)	0 (0)	0 (0)	2 (33.3)	0 (0)	1 (33.3)	1 (14.3)
Mean radiosensitivity values B/M	0 Gy (SD)	0.033 (+/- 0.053)	0.028 (+/- 0.035)	0.015 (+/- 0.014)	0.037 (+/- 0.064)	0.029 (+/- 0.044)	0.008 (+/- 0.011)	0.019 (+/- 0.019)	0.023 (+/- 0.032)	0.033 (+/- 0.040)	0.021 (+/- 0.018)	0.035 (+/- ---)	0.035 (+/- 0.036)	0.000 (+/- ---)	0.027 (+/- 0.036)	0.005 (+/- 0.009)	0.059 (+/- 0.045)	0.104 (+/- 0.157)
	2 Gy (SD)	0.488 (+/- 0.134)	0.443 (+/- 0.155)	0.590 (+/- 0.096)	0.507 (+/- 0.137)	0.490 (+/- 0.124)	0.446 (+/- 0.133)	0.431 (+/- 0.082)	0.513 (+/- 0.061)	0.431 (+/- 0.096)	0.567 (+/- 0.155)	0.424 (+/- ---)	0.432 (+/- 0.128)	0.510 (+/- ---)	0.548 (+/- 0.120)	0.581 (+/- 0.064)	0.544 (+/- 0.146)	0.500 (+/- 0.108)

### Fluorescence in situ-hybridization assay (FiSH)

**Fig. 1:**
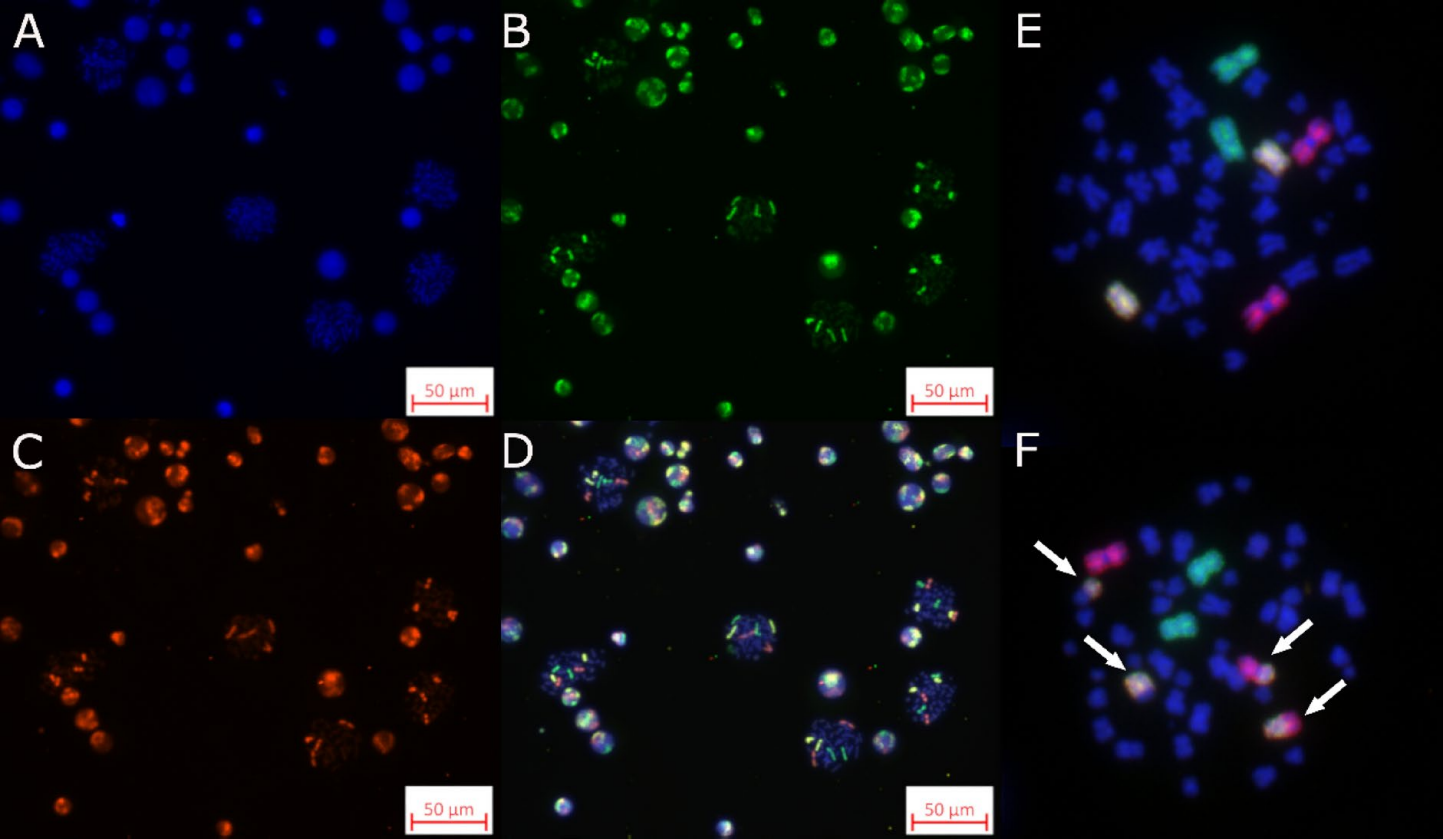
Painting of chromosomes 1, 2 and 4 with FiSH for the analysis of chromosomal aberrations. Fluorescence-stained chromosomes in metaphase with 400 x magnification (**A**–**D**) and 630 x magnification (**E**, **F**). (**A**) DAPI filter depicting the DNA of cells; (**B**) FITC filter showing chromosomes #2 and #4; (**C**) Cy3 filter depicting chromosomes #1 and #4; (**D**) multicolor filter showing chromosome #1 in red, #2 in green, and #4 in yellow. (**E**) Metaphase without chromosomal aberrations. Chromosome #1 stained red, #2 green and #4 yellow. (**F**) Two translocations resulting in a total of 4 breaks. Translocation from #1 (red) to #4 (yellow). Translocation from #4 (yellow) to another chromosome in DAPI (blue). Translocations are marked by arrows.

Three-color fluorescence in situ hybridization (FiSH) was used to measure radiosensitivity. Approximately 8 milliliters of venous blood was collected from each patient in heparin cubes (NH4-heparin, Sarstedt, Nürnbrecht, Germany). The blood samples were separated and one of them was placed in an acrylic block to be irradiated using a 6 MV linear accelerator delivering 2 Gy (Versa, Electa, Sweden). This dose correlates with a fractionated daily dose used in the radiotherapy of cancer patients^15,16^. The unirradiated sample is used to quantify the background of existing chromosomal aberrations. The 0 Gy and 2 Gy blood samples were each incubated for 48 h at 37 °C in a cell culture medium containing Roswell Park Memorial Institute Medium (RPMI), 2.5% phytohemagglutinin (Biochrom, AG, Berlin, Germany), 1% penicillin/streptomycin and 15% fetal calf serum^14^. Phytohemagglutinin was used to stimulate T-lymphocyte division. Cells were arrested in metaphase by adding a 0.1 µg/mL colcemid solution (Gibco, Waltham, MS, USA). The blood culture was then processed for lymphocyte extraction by repeated cycles of centrifugation and addition of acetic acid-methanol fixative. A 0.4% potassium chloride solution was used to swell the cells during incubation at 37 °C for 20 min. The cells were then dropped onto slides, which were further prepared for hybridization with chromosome-specific probes. Fluorescent probes were used to stain the three largest chromosomes 1, 2 and 4 in red, green, and yellow respectively. These chromosomes represent 22% of the total DNA. DAPI (4′,6-diamidin-2-phenylindol) was used for counterstaining all chromosomes^17^. After performing a post-wash, mitoses on the slide were covered with a coverslip using Vectashield (Newark. CA. USA). Finally, the slides were visualized by a fluorescence microscopy (Zeiss, Axio Imager Z2, Göttingen, Germany). The entire procedure followed a standard protocol (Fig. 1)^18^.

### Image analysis

Painted chromosomes in the metaphases were acquired by a fluorescence microscopy (Zeiss, Axio Imager Z2, Göttingen, Germany) at 630x magnification using Metasystems software (Metapher 4 V3.10.1, Altlussheim, Germany). First, the metaphases were automatically detected at 100x magnification for position finding. A color image of each metaphase was captured at 630x magnification. The resulting images were then used to detect chromosomal aberrations using image analysis software (Biomas, Erlangen, Germany). The software enables manual analysis of the images and transfers the number of breaks in each image to an Excel spreadsheet (Excel, Microsoft Corporation, Redmond, WA, USA). The aim was to analyze at least 100 metaphases at 0 Gy and 200 at 2 Gy if possible. On average, 116 metaphases were reached at 0 Gy and 227 metaphases at 2 Gy.

Different types of chromosomal breaks were counted and scored according to Savage and Simpson^19^ based on the number of DNA double-strand breaks required to cause an aberration of the particular type. Breaks and deletions were counted as one break, translocations, dicentrics and rings as two breaks and insertions as three breaks. For complex aberrations, the minimum number of breaks theoretically required for that appearance was counted. This results in a value of breaks per metaphase, which is then used as a measure of radiosensitivity (B/M). The final value is the 2 Gy value, from which the background has been subtracted. This yields the pure influence of the irradiation, independent of any pre-existing aberrations. Therefore, the corrected value of 2 Gy will be independent of the background aberrations^20^. The cut-off for increased radiosensitivity was set at 0.5 B/M based on existing studies showing increased radiosensitivity above this level^21–24^.

### Statistics

Graph Pad Prism (GraphPad Software, San Diego, California, USA) and Excel (Microsoft Corporation, Redmond, Washington, USA) were used for statistical calculations and data visualization. T-test (*n* > 30) or non-parametric Man-Whitney U-test (*n* < 30) was used to compare the different cohorts for significant differences. A binomial distribution was used to calculate the expected probability of gene mutation clusters within the respective gene domains. Pearsons r was used to test for correlations. The significance level was set at 5%.

## Results

### Chromosomal radiosensitivity of risk genes

**Fig. 2:**
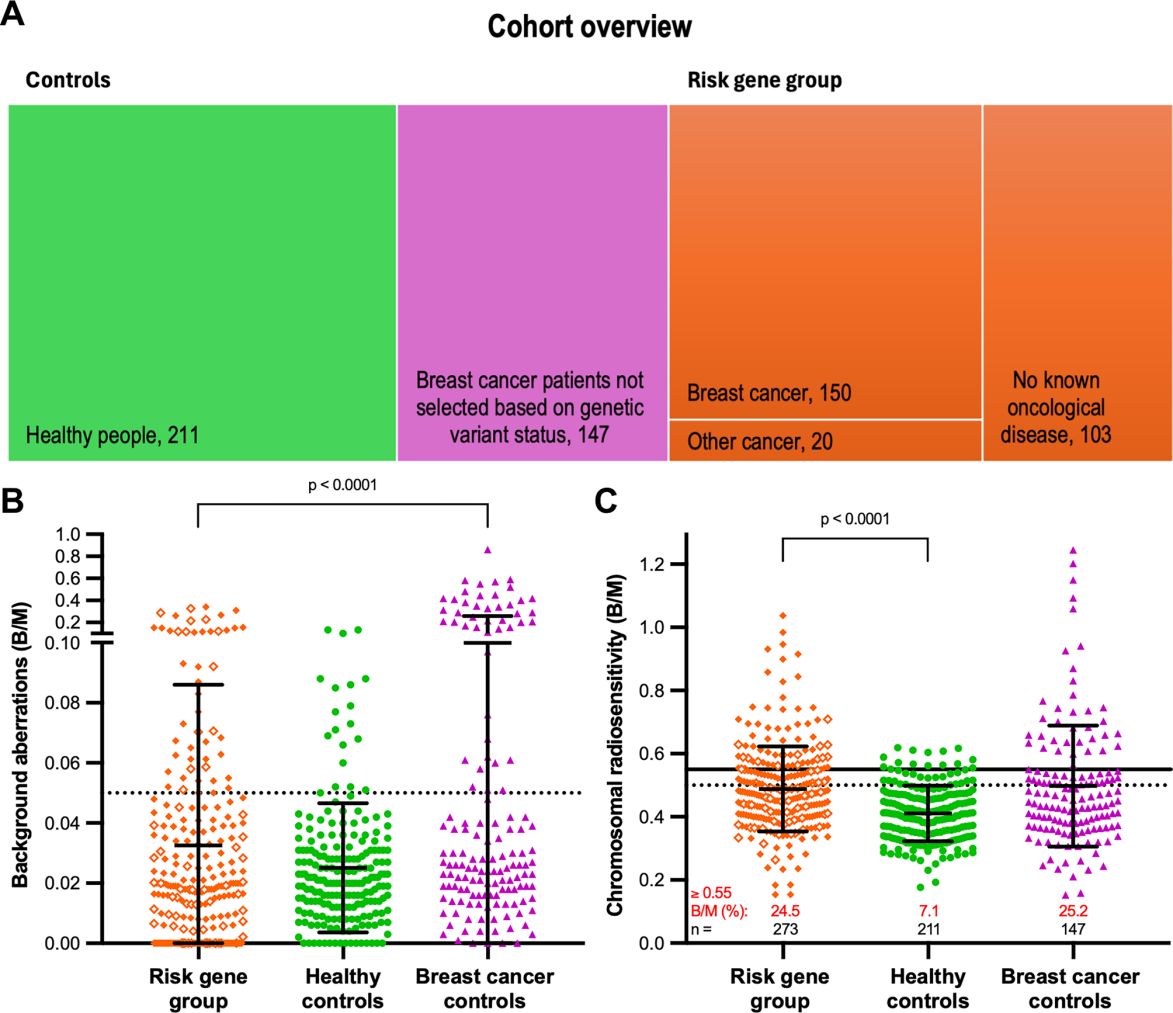
Comparison of the breast cancer risk gene cohort with breast cancer controls and healthy controls. (**A**) Cohort overview as a treemap. (**B**) Background aberrations (0 Gy). Cut-off value is marked for ≥ 0.05 B/M, representing increased background aberrations. (**C**) Chromosomal radiosensitivity (2 Gy). Cut-offs are marked for ≥ 0.5 B/M (dotted line) and ≥ 0.55 B/M (solid line) for increased radiosensitivity and the cut-off for dose reduction recommendations. Proportions (%) of risk genes reaching values ≥ 0.55 B/M are written in red for each column. Statistically significant differences are indicated by p values above each graph. Black lines with bars show the mean radiosensitivity values (B/M) with standard deviation (SD) for each cohort. The number of patients analyzed in each column is given below (n). Open diamond symbols indicate that the data were derived from a previous publication.

The risk gene group, comprising 273 people with breast cancer risk genes, was compared with healthy controls and breast cancer controls for whom non-carrier status had not been confirmed, which may limit conclusions relating to specific genes. However, the proportion of undetected variant carriers expected in these control groups is likely to be low. (Fig. 2A). Background and radiosensitivity were determined by the presence of chromosomal aberrations (B/M) before and after ex vivo irradiation. Background aberrations in the risk gene group (0.033 B/M ± standard deviation 0.053) were not significantly different to healthy controls (0.025 B/M ± 0.022) (*p* = 0.055). Background aberrations in the breast cancer controls (0.100 B/M ± 0.159) were higher than in the risk gene group (*p* < 0.001). The risk gene group contains a higher proportion of background aberration values ≥ 0.05 B/M (17.9%) compared to healthy controls (9.0%). Breast cancer controls had the highest proportion ≥ 0.05 B/M (30.6%) (Fig. 2B).

The chromosomal aberrations were determined after irradiation and subtraction of the background to yield radiosensitivity. The chromosomal radiosensitivity of the risk gene group (0.488 B/M ± 0.134) was clearly higher than that of healthy controls (0.411 B/M ± 0.088) (*p* < 0.001) and was nearly equivalent to that of breast cancer controls (0.498 B/M ± 0.192) (*p* = 0.563). 24.5% of risk gene carriers had values ≥ 0.55 B/M like the breast cancer control group (25.2%), whereas in the healthy controls this cut-off was reached only by fewer individuals (7.1%) (Fig. 2C).

**Fig. 3:**
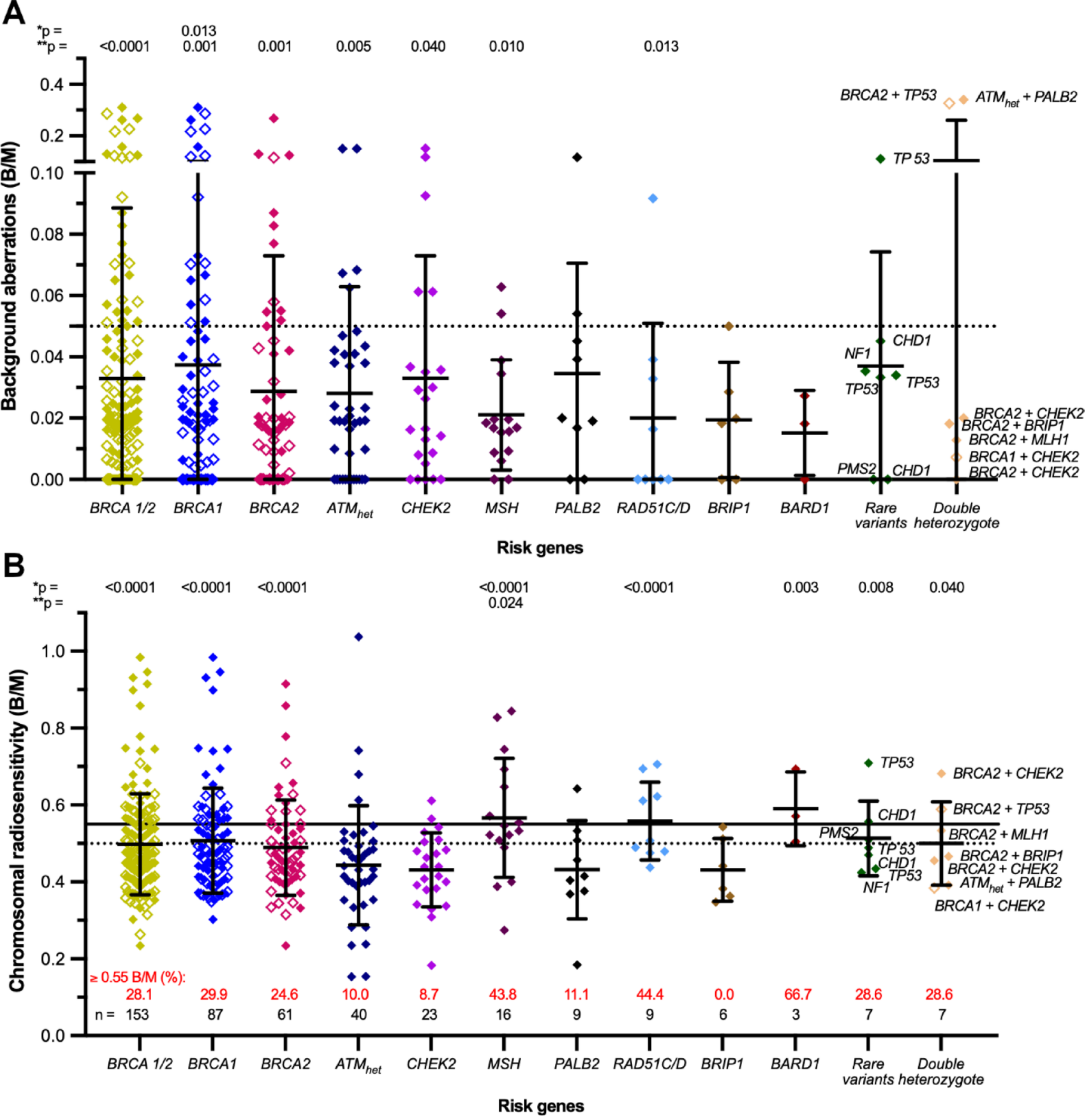
Analysis of background aberrations and chromosomal radiosensitivity in patients carrying individual breast cancer risk genes. (**A**) Background chromosomal aberrations, expressed as B/M, were studied in nine breast cancer risk gene groups, including one group with rare variants and another with two variants of the breast cancer gene. Cut-off value is marked for ≥ 0.05 B/M, representing increased background aberrations. (**B**) Chromosomal aberrations induced by 2 Gy after background subtraction, indicating the radiosensitivity of nine breast cancer risk gene groups, including one group with rare variants and another with two variants of the breast cancer gene. The cut-offs are marked at ≥ 0.5 B/M (dotted line) and ≥ 0.55 B/M (solid line) to indicate increased radiosensitivity, as well as the cut-off point for dose reduction recommendations. The number of individuals analyzed in each group is indicated below (n). The percentages (%) of risk genes that reach values ≥ 0.55 B/M are shown below in red for each group. Black lines with bars show the mean radiosensitivity values (B/M) with standard deviation (SD) for each cohort. Statistically significant differences are indicated by p-values above each diagram in comparison to healthy control subjects in the row marked *p and to breast cancer patients in the row marked **p. Open diamond symbols indicate that the data were derived from a previous publication.

The next question was whether specific genes within the group of cancer risk genes make individuals particularly sensitive to radiation. Among the different breast cancer risk genes, only *BRCA1* (0.037 B/M ± 0.063) and *TP53* (0.059 B/M ± 0.045) had higher background rates compared to healthy controls (*p* = 0.013 and *p* = 0.032, respectively). The majority of risk genes exhibited considerably lower values in terms of background aberrations when compared to breast cancer controls. These were *BRCA1/2* (*p* < 0.001), *ATM*_*het*_ (*p* = 0.005), *CHEK2* (*p* = 0.040), *RAD51* (*p* = 0.013), and MSH (*p* = 0.010). Only patients with double heterozygotes had mean background aberration values (0.104 B/M ± 0.157) that were similar to those of the breast cancer control group. The highest proportion of individuals with a B/M ratio of at least 0.05 was found in *BRCA1/2* (20.7% and 19.7%) and *PALB2* (22.2%), *CHEK2* (21.7%) and double heterozygote group (28.6%) (Fig. 3A).

Chromosomal radiosensitivity in the individual risk genes were only significantly increased in *MSH* (0.567 B/M ± 0.155) (*p* = 0.024) compared to breast cancer patients and highly increased compared to healthy controls (0.411 B/M ± 0.088) (*p* < 0.0001). Chromosomal radiosensitivity was increased in *BRCA1/2* combined (0.498 B/M ± 0.131) and *BRCA1* (0.507 B/M ± 0.137) or *BRCA2* (0.489 B/M ± 0.124) (*p* < 0.0001). Some groups were less elevated such as *ATM*_*het*_ (0.443 B/M ± 0.155), *BRIP1* (0.431 B/M ± 0.082), *CHEK2* (0.431 B/M ± 0.096) and *PALB2* (0.432 B/M ± 0.128). Double heterozygote variants (0.500 B/M ± 0.109) (*p* = 0.040) were slightly elevated. Rare variants (0.513 B/M ± 0.097) (*p* = 0.008), *RAD51C/D* (0.559 B/M ± 0.102) (*p* < 0.0001), *MSH* (0.567 B/M ± 0.155) (*p* < 0.0001) and *BARD1* (0.590 ± 0.096) (*p* < 0.003) were extremely elevated.

An important factor in assessing radiosensitivity is determining the proportion of individuals for whom a dose reduction could be considered, starting with a value of 0.55 B/M for radiotherapy. This proportion was high for *BRCA1/2* (28.1%), rare variants (28.6%) and double heterozygote (28.6%) and therefore comparable to the breast cancer group (25.2%) and much higher than in the healthy cohort (7.1%). It was very high for *MSH* (43.8%), *RAD51C/D* (44.4%), and *BARD1* (66.7%) (Fig. 3B).

### Radiosensitivity among the non-oncological and oncological risk gene carriers

**Fig. 4:**
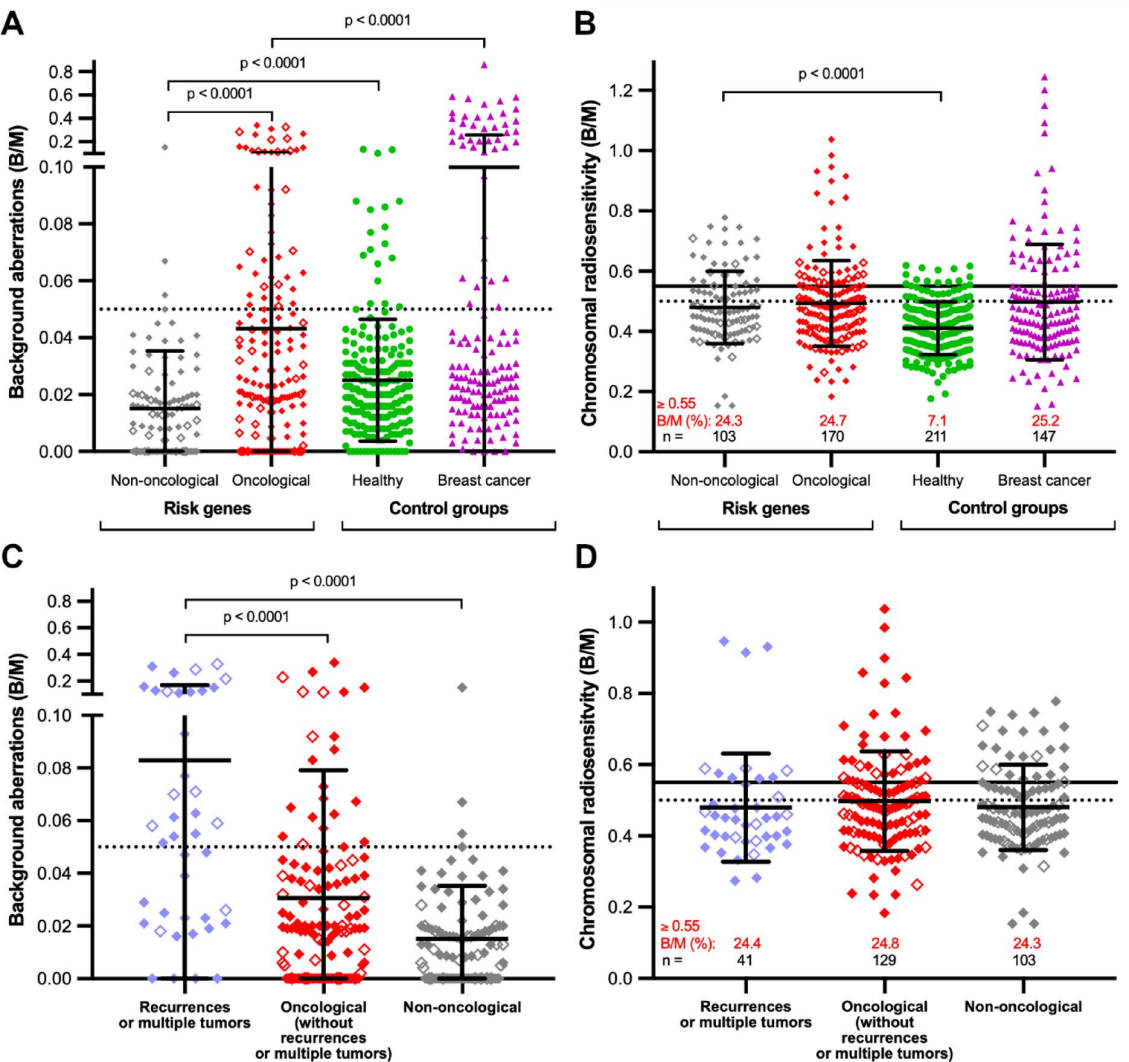
Influence of oncological disease on increased radiosensitivity. Comparison of risk genes separated into non-oncological and oncological subgroups as well as the control groups healthy controls and breast cancer patients for (**A**) background values and (**B**) chromosomal radiosensitivity. Comparison of risk gene patients with recurrence and multiple tumors with oncological and non–oncological risk gene carriers for (**C**) background values and (**D**) chromosomal radiosensitivity. Black lines with bars show the mean radiosensitivity values (B/M) with standard deviation (SD) for each cohort. The number of patients analyzed in each column is given below (n). Cut-off value is marked for ≥ 0.05 B/M representing increased background aberrations (**A**, **C**). The dotted line indicates the cut-off for increased radiosensitivity (≥ 0.5 B/M), and the solid line indicates the cut-off for dose reduction recommendations (≥ 0.55 B/M) (**B**, **D**). Statistically significant differences are indicated by p values above each graph between non-oncological and oncological, non-oncological and healthy controls, oncological and breast cancer controls (**A**, **B**) and equally between recurrences/multiple tumors compared to oncological and non-oncological (**C**, **D**). Open diamond symbols indicate that the data were derived from a previous publication.

One possibility is that patients with increased radiosensitivity are also at an increased risk for cancer disease, meaning that radiosensitivity is higher in this group. Therefore, we studied the association between oncological disease and increased radiosensitivity. The background aberrations were overall clearly higher in the oncological group (0.043 B/M ± 0.064) than in the non-oncological group (0.015 B/M ± 0.020) (*p* < 0.0001), though they were lower than in the group of healthy controls with unknown variant status (0.025 B/M ± 0.0215) (*p* < 0.0001). Oncological risk gene carriers had also lower background aberrations compared to breast cancer control group with unknown variant status (0.100 B/M ± 0.159) (*p* < 0.0001) (Fig. 4A).

There was no difference in chromosomal radiosensitivity between non-oncologic (0.480 B/M ± 0.120) and oncologic (0.493 ± 0.142) risk gene carriers (*p* = 0.445). Non-oncological patients were more radiosensitive than healthy controls (0.411 B/M ± 0.088) (*p* < 0.0001). There was no difference between oncological patients and breast cancer controls (0.498 B/M ± 0.192) (*p* = 0.419) (Fig. 4B). We were also interested in whether more severe diseases in the sense of diseases with recurrences or multiple tumors have an influence on radiosensitivity. Background aberrations (0.083 B/M ± 0.086) in these patients were higher than in oncological risk gene carriers without recurrence or multiple tumors (0.031 B/M ± 0.049) (*p* < 0.0001), as well as in non-oncological patients (0.015 B/M ± 0.020) (*p* < 0.0001) (Fig. 4C). There was no difference in chromosomal radiosensitivity after irradiation between the three risk gene carrier groups: the recurrence group (0.479 B/M ± 0.152), the oncological group without recurrences (0.497 B/M ± 0.140) and the non-oncological group (0.480 B/M ± 0.120) respectively (*p* = 0.483 and *p* = 0.973) (Fig. 4D).

### Radiosensitivity based on location or type of *BRCA1/2* variants

The range of radiosensitivity values for the two risk genes, *BRCA1* and *BRCA2*, is broad, spanning from 0.234 B/M to 0.984 B/M (Fig. 3A,B). We were interested in whether the location and type of variant of both genes influences radiosensitivity and can explain these intra-cohort differences. We first compared the expected occurrence probability with the observed probability of occurrence. *BRCA1* gene mutations were more frequent in the ring domain but much more frequent than expected in the BRCT domain. In the *BRCA2* gene, mutations in the N-terminal region (1–1001 aa) and the DNA-binding domain were overrepresented (Table 2). Then, we studied whether mutations in specific domains cause increased radiosensitivity.

**Table 2:** The frequency and distribution of expected and observed mutations in the *BRCA1* (67 mutations) and *BRCA2* (51 mutations) genes.

	67 mutations	N-terminus (aa)	Length (aa)	Expected mutations (probability)	Observed mutations (probability)	Factor observed/expected
BRCA1	RING domain	1–109	109	4; (0.201)	7; (0.055)	1.8
	Interdomain region	110–1279	1170	42; (0.100)	28; (< 0.001)	0.7
	Serine cluster domain (SCD)	1280–1524	245	9; (0.141)	5; (0.061)	0.6
	Interdomain Region	1525–1649	125	4; (0.195)	2; (0.109)	0.5
	BRCT domains	1650–1863	214	8; (0.148)	25; (< 0.001)	3.1

	51 mutations	N-terminus (aa)	Length (aa)	Expected mutations (p =)	Observed mutations (p =)	Factor observed/expected
BRCA2	Interdomain regions	1–1001	1001	15; (0.118)	17; (0.099)	1.1
	BRC repeats	1002–2085	1084	16; (0.119)	10; (0.019)	0.6
	Interdomain regions	2086–2481	396	6; (0.168)	4; (0.135)	0.7
	DNA binding domain	2482–3184	703	11; (0.137)	20; (0.001)	1.9
	Interdomain regions	3185–3262	78	1; (0.366)	0; (0.305)	0.0
	RAD51-binding domain	3263–3385	123	2; (0.291)	0; (0.151)	0.0

The expected frequency was calculated by evenly distributing the mutations according to the proportional length of the different domains. Then, the probability of the calculated and observed frequencies of variants in the different domains was calculated using a binomial distribution. A low probability indicates that the observed or expected frequency is unexpected.

**Fig. 5:**
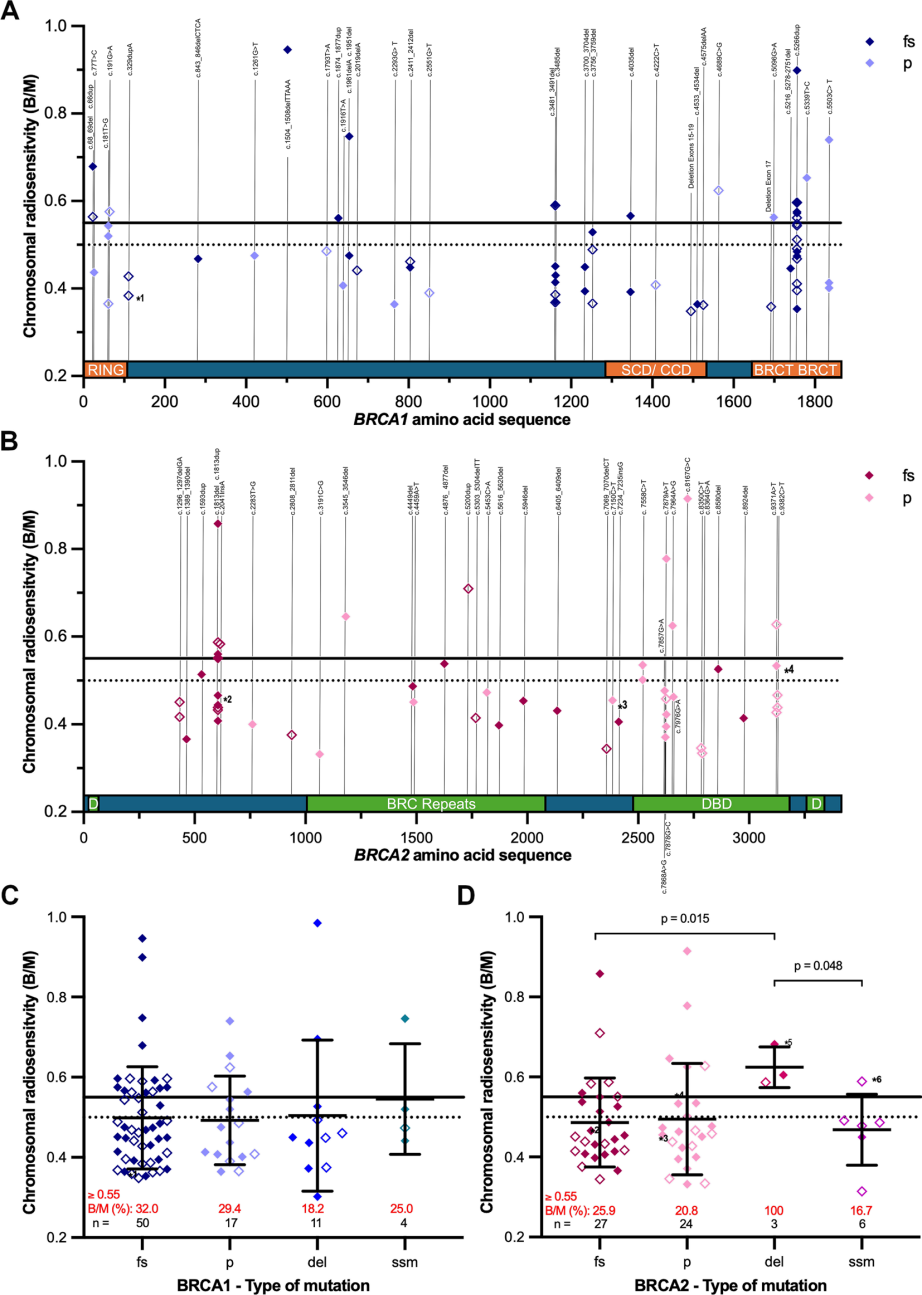
Chromosomal radiosensitivity depending on the location and type of variants in the *BRCA1* and *BRCA2* gene. Chromosomal radiosensitivity in dependence of amino acid sequences of (**A**) 67 *BRCA1* gene variants and (**B**) 51 *BRCA2* gene variants including the relevant binding domains for DNA repair process^25,26^. Variant types (**C**) in the *BRCA1* gene and (**D**) in the *BRCA2* gene. Black lines with bars show the mean radiosensitivity values (B/M) with standard deviation (SD) for each cohort. The number of patients analyzed in each column is given below (n). Cut-offs are marked for ≥ 0.5 B/M (dotted line) and ≥ 0.55 B/M (solid line) for increased radiosensitivity and the cut-off for dose reduction recommendations. The vertical lines represent mutations at the cDNA level. Statistically significant differences are indicated by p values above each graph. fs: frameshift, p: point variants, del: deletion, ssm: splice-site variants. Double heterozygote variants are marked: *1 *BRCA1* + *CHEK2*, * 2 *BRCA2* + *BRIP1*, *3 *BRCA2* + *CHEK2*, *4 *BRCA2* + *MLH1*, *5 *BRCA2* + *CHEK2*, *6 *BRCA2* + *ТР53*. Open diamond symbols indicate that the data were derived from a previous publication.

Of the 67 subjects who underwent genetic testing for the presence of the *BRCA1* gene, 21 (31.3%) had a B/M value of ≥ 0.55. Frameshift variants were present in 16 out of 50 patients (32.0%) with values of ≥ 0.55 B/M, while point variants were present in five out of 17 patients (29.4%). Increased radiosensitivity accumulates in critical protein-binding regions, including the RING domain (aa 1–109) and the BRCT domain (aa 1650–1863). In the RING domain, three out of seven patients (42.9%) had a value of ≥ 0.55 B/M, and in the BRCT domain, this figure was 11 out of 25 patients (44%) (Fig. 5A). The proportion of patients reaching values of at least 0.55 B/M in the *BRCA2* gene was 12 out of 51 (23.5%). Frameshift variants accounted for seven out of 27 patients (25.9%), while point variants accounted for five out of 24 patients (20.8%). Accumulation in the binding domain was found in four out of 20 patients (20%) in the DBD domain (~ aa 2482–3184). Five out of ten (50%) individuals with a frameshift variant affecting amino acid (aa) 605 in the *BRCA2* protein reached values of at least 0.55 B/M. Among amino acids 1–1001, the ratio of ≥ 0.55 B/M was observed in six of the 17 (35.3%) patients (Fig. 5B).

No differences in radiosensitivity were found when comparing the different types of variants in the *BRCA1* gene (Fig. 5C). Three out of three *BRCA2* gene deletions reached the cutoff value of ≥ 0.55 B/M and were more radiosensitive overall (0.624 B/M ± 0.051) than frameshift variants (0.486 B/M ± 0.111) (*p* = 0.015) and splice site variants (0.468 B/M ± 0.089) (*p* = 0.048) (Fig. 5D).

### Radiosensitivity changes with age

**Fig. 6:**
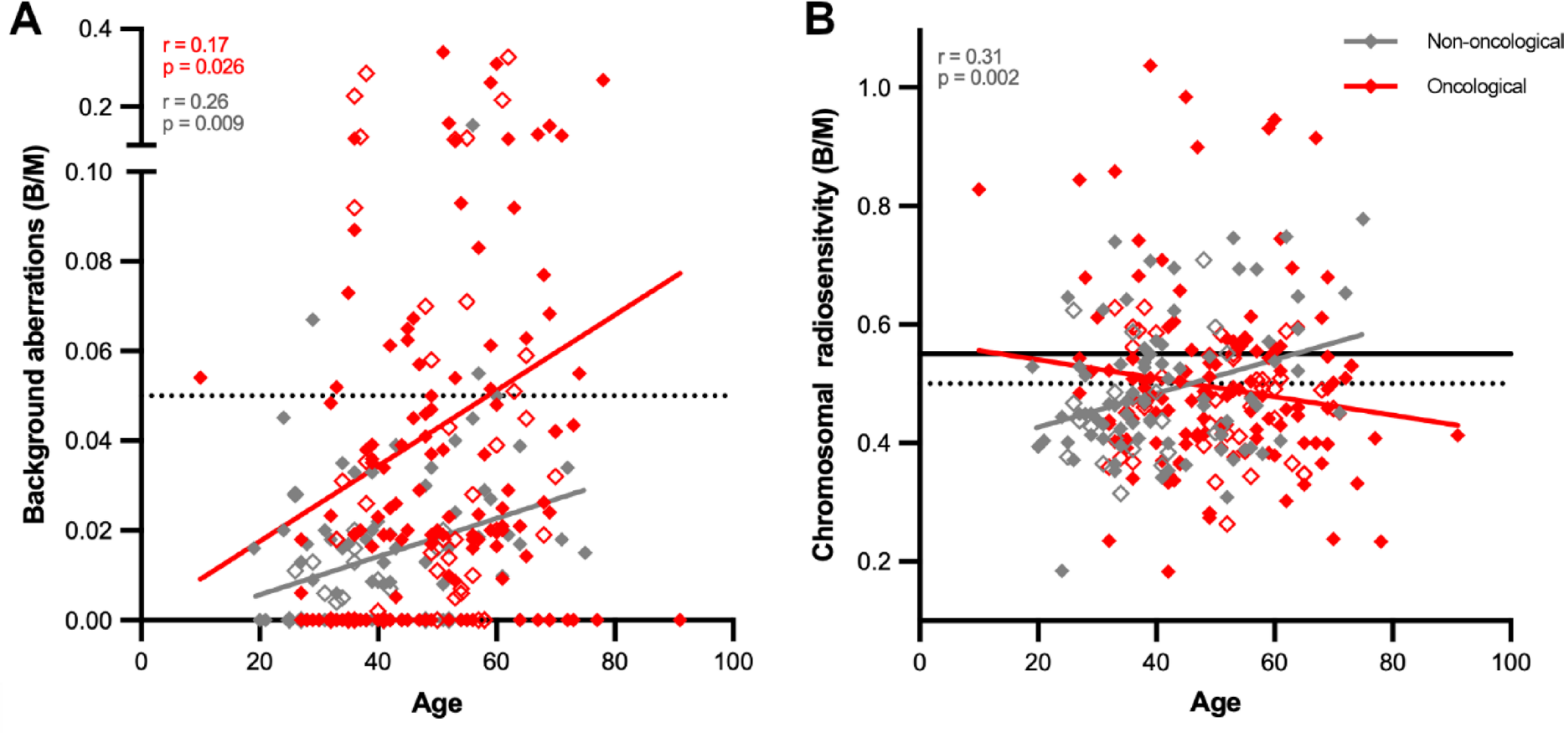
Association of radiosensitivity with age of the risk gene cohort. (**A**) Background aberrations with age and (**B**) chromosomal radiosensitivity with age. The diamond symbols depict oncological patients as red and non-oncological patients as grey. The lines are fitted using linear regression. Statistically significant differences are shown above each diagram with Pearsons r and p – values. Cut-offs are marked for ≥ 0.05 B/M in (**A**) as well as ≥ 0.5 B/M (dotted line) for increased radiosensitivity and ≥ 0.55 B/M (solid line) as the cut-off for dose reduction recommendations (**B**). Open diamond symbols indicate that the data were derived from a previous publication.

Next, we were interested in whether there was an association between radiosensitivity and age. We studied the radiosensitivity in the risk gene cohort in relation to the age separately considering the presence of an oncological disease. In both groups background aberrations were weakly positively correlated with increasing age (oncological: *r* = 0.17, *p* = 0.026 and non-oncological: *r* = 0.26, *p* = 0.009) (Fig. 6A). Individuals with oncological diseases (0.0008 B/M/year) had nearly double the increase in background aberrations compared to individuals without oncological diseases (0.0004 B/M/year).

Chromosomal radiosensitivity in the non-oncological group (*r* = 0.31, *p* = 0.002) correlated weakly positive with an increase around 0.003 B/M/year. There was no association in the oncological risk gene carriers (*r* = −0.14, *p* = 0.066) (Fig. 6B).

## Discussion

### Classification of results

The study’s findings suggest that individuals with breast cancer risk genes do not differ significantly from breast cancer patients with unknown variant status. However, non-oncological patients with risk genes are more sensitive to radiation than healthy individuals without known variant status, which can certainly be explained by the prevalence of risk gene variants. Overall, patients with cancer risk genes exhibit slightly increased mean sensitivity to radiation, a characteristic that differs from that observed in the healthy control group. Significant differences in radiosensitivity exist among various cancer risk genes. Patients with *CHEK2*, *BRIP1*, *PALB2*, or *ATM*_*het*_ have relatively average radiosensitivity, similar to healthy individuals. In contrast, variants in *BRCA1/2* and *TP53* lead to a limited increase in radiosensitivity, comparable to that observed in breast cancer patients. Patients with variants in *MSH*, *RAD51C/D*, and *BARD1* lead to a significantly higher mean radiosensitivity than in control groups. It must be considered that the interpretation of these findings is limited by the absence of confirmed non-carrier status in the control groups and results for some genes should be interpreted with caution due to the small size of the cohort.

However, it should be noted that radiosensitivity can vary greatly within a gene variant. Even among genes with an average low radiosensitivity, there are individuals with high radiosensitivity. Similarly, among the three genes with very high radiosensitivity, there are individuals with average sensitivity. Thus, the correlation between measured radiosensitivity should be viewed in this context. An increase in chromosomal aberrations starting from about 0.4 B/M results in an ever-increasing risk of an undesirable therapeutic outcome up to a value of approximately 0.8 B/M. This range must be regarded as stochastic. Beyond that point, a more deterministic range begins, where the risk of an undesirable outcome is very high^27^.

Even genes with diverse functions may contribute to radiation sensitivity due to their role in DNA damage response pathways. *CHEK2* and *TP53* are checkpoint control proteins; *ATM* and *BRCA1* are DNA repair sensor proteins; and *BRCA2*, *PALB2*, and *BARD1* form heteromers that participate in DNA double-strand break (DSB) repair through homologous recombination (HR). *BRCA1* and *RAD51* are key enzymes in HR^7^. MSH, on the other hand, is a protein involved in mismatch repair^28^.

A specific mutation probably cannot be used to predict whether there is an increased sensitivity to radiation. This becomes clear when considering variant c.5266dupC (p.Gln1756fs) of *BRCA1*. This variant was studied in 18 patients, and the measured radiosensitivity ranged from 0.35 to 0.90 B/M. This shows a large variation in radiosensitivity within a specific mutation. Radiosensitivity is a complex, multifactorial process requiring many modifying factors to produce such a wide range of variation, as illustrated here^29^.

More mutations are found in the RING and BRCT binding domains of *BRCA1*, yet much less in the serine cluster domain (SCD)^12^. These two overrepresented domains are associated with increased radiosensitivity, but not the SCD domain. It is unclear why carriers of variant genes have these mutations more frequently in the binding domains. However, we believe that these mutations likely cause increased radiosensitivity because the loss of the ability to bind to other proteins results in a lack of repair activity^30^.

The main reason for measuring the background level of chromosomal aberrations is to subtract it from the level induced by radiation to obtain only the aberrations caused by radiation^12^. However, it is also exciting because different groups have different numbers of chromosomal aberrations as a background. Since ionizing radiation is rare in everyday life, other toxins are more likely responsible for these background mutations. *BRCA1/2* plays a role in repairing DNA double-strand breaks via homologous recombination. More importantly, it is involved in the Fanconi pathway for repairing DNA interstrand crosslinks. These crosslinks are induced by chemicals^31^. The higher proportion of aberrations in patients with oncological disease, especially those with multiple tumors and recurrences, likely reflects their higher exposure to carcinogens by diet and the environment. Furthermore, genetic variants in enzymes that metabolise carcinogens, such as cytochrome P450 (CYP), glutathione S-transferases (GST) and N-acetyltransferases (NAT), may alter the metabolism of environmental carcinogens, affecting cancer risk as a result^32^. Additionally, different mean age could explain this difference as background aberrations increase in older people^33^, and risk gene carriers with tumor disease were on average about 10 years older than non-oncological patients. In the risk gene group, we found slightly increasing radiosensitivity with age for the non-oncological patients and no association with age in the oncological patients^13^. Maybe a selection bias leads to that result, because among the limited number of individuals especially young patients were studied for their radiosensitivity.

### Consequences of radiotherapy

The most common side effects after breast irradiation are dermatitis, fatigue or pain. The design of radiotherapy plays an important role. Patients who received a 40 Gy/15 fractions regimen (three-week RT) experienced less toxicity than those who received 50 Gy/25 fractions (five-week RT)^34^. A study found that partial breast irradiation in lower-risk patients reduced symptoms such as pain, systemic side effects and breast symptoms^35^. In addition, techniques such as deep inspiration are being employed in studies to minimize radiation exposure to the heart. Cardiotoxicity is a late effect of radiation therapy, particularly in cases of left-sided breast cancer^36^. Serious side effects after breast irradiation are rare due to the lower total dose (~ 50 Gy)^37^ compared to, for example, head and neck radiotherapy (~ 68 Gy)^38^. In the ABPI2 study, only one patient out of 170 who received accelerated partial breast irradiation had grade 2 pneumonitis. But this patient had also an elevated radiosensitivity value of 0.79 B/M probably due to vitiligo, which has already been discussed to cause higher radiosensitivity^39^. Dose reduction could be considered in patients ≥ 0.55 B/M because of the increased risk of side effects, and we recommend strict follow-up for these patients. In the total risk gene group, we identified 67 (24.5%) patients with radiosensitivity values ≥ 0.55 B/M, for whom we would recommend these adjustments^27^. Since radiation side effects are rare in breast cancer patients, we could only find two patients with radiodermatitis (0.510 B/M, 0.745 B/M) and one patient with radio colitis (0.450 B/M) documented in the risk gene group.

### Reasons for radiosensitivity in heterozygosity and application of radiosensitivity knowledge

The question arises as to why heterozygous variants can lead to increased radiosensitivity since one allele is unaffected. For cancer development, the second hit theory involving loss of heterozygosity (LOH) is an important aspect. In addition. studies suggest other causes without LOH. One explanation for a non-functional protein is haploinsufficiency, which could result in a reduced amount of protein that is unable to fully perform its function. This is accentuated under stress by metabolic disorders, DNA damage and in tissues with high proliferation and cell division, such as breast or ovarian tissue^40–42^. Whereas in other genes, like *TP53*, the dominant negative effect of a mutated subunit in the protein causes cancer^43^. This may explain the inter-individual differences in radiosensitivity found in risk gene carriers due to different reasons for the dysfunctional protein.

With a total of 273 high-risk individuals, the study represents a comparatively large overall cohort. The *BRCA1/2* subgroup is particularly large, allowing for more robust conclusions to be drawn. In contrast, the numbers for other gene subgroups remain limited (e.g. *BARD1*,* RAD51C/D*). The comparison groups included 147 breast cancer patients and 211 healthy individuals. However, as the controls are not confirmed non-carrier groups, the validity of causal conclusions regarding gene-specific effects is limited. Therefore, while the dataset enables meaningful analyses of general radiation sensitivity and *BRCA1/2*-associated effects, conclusions regarding rarer gene variants should be considered exploratory and preliminary. DNA damage, as measured by the FiSH method, correlates with radiation exposure. It is also a suitable long-term biomarker for mapping radiation damage^44^. Chromosomal aberrations by FiSH are suitable for predicting radiosensitivity because the value of B/M corresponds to the clinical side effects of radiotherapy^45,46^. Even if the effects of radiation in breast cancer therapy are minimal, the knowledge gained about the radiosensitivity of breast cancer risk gene carriers could be therapeutically useful. In theory, radiosensitivity values found in lymphocytes can be extrapolated to the tumor. This could make it possible to predict the tumor-reducing effect of radiotherapy. Radiotherapy could be individually optimized to a lower, less harmful dose, trying to balance efficacy with minimized side effects^27^. For the genes with increased radiation sensitivity identified in this study (*BARD1*, *RAD51C/D* and *MSH*), current recommendations do not indicate any contraindications for radiation therapy. Radiation therapy is not recommended in cases of *TP53* mutations. In addition, there are no restrictions for *BRCA1/2*, though mastectomy is often performed for therapeutic reasons due to the risk of recurrence^47^.

### Limitations

There are some limitations in this study. For some rare variants, including *BARD1*,* BRIP1*,* CHD1*,* NF1*,* PALB2*,* PMS2*,* RAD51C/D*,* TP53* and double heterozygote variants, relatively few patients could be analyzed. The results of these subgroups should be considered exploratory rather than definitive and require confirmation or refutation in larger studies. The comparison groups are limited due to the unknown proportion of risk carriers and the absence of confirmed non-carrier status, which results from the historical collection of data for a study with a different focus. Therefore, gene-specific conclusions are limited in this study, as it cannot be excluded that a small proportion of mutation carriers may be present in the comparison group. It would be valuable for future studies to include a control cohort with confirmed non-carrier status to address this limitation. The breast cancer group has many outlying values > 0.1 B/M in background aberrations compared to other groups. This could have been caused by previous cancer treatment. Background levels of heterogeneity may introduce bias through subtraction, which could potentially influence the final radiosensitivity results. Several factors can influence radiosensitivity. In addition to variant status, which affects DNA repair mechanisms, other genetic modifying variables are present. Other factors that influence radiosensitivity should be considered, such as age, which increases slightly with advancing age, and inflammatory diseases, such as rheumatoid arthritis^48^, vitiligo^39^ or lupus erythematosus^27^ and drugs like chloroquine and kinase inhibitors^49^. However, no gender-related differences were found^50^. As several variables can influence radiation sensitivity, it would have been preferable to conduct multivariate regression analyses to capture these interactions more comprehensively and arrive at more nuanced conclusions by taking multiple influencing factors into account simultaneously. This should be considered in future studies involving larger subgroups. Additionally, the FiSH radiosensitivity test should be performed prior to radiotherapy, as high background rates can complicate data interpretation^51^.

## Conclusion

Different gene variants exhibit significantly different radiation sensitivities. Additionally, interindividual radiation sensitivities within a gene variant can vary greatly. *BRCA1* and *BRCA2* carriers are slightly more sensitive to radiation. There is a trend toward increased radiosensitivity in patients with variants in the *RAD51C/D*,* MSH*, or *BARD1* genes. Dose reduction could be considered for these patients undergoing radiotherapy. Performing radiosensitivity testing would be very useful for identifying these patients, predicting their risk of experiencing side effects, and enabling personalized dose adjustments for radiotherapy.

## Data Availability

The generated data is available in the main text. Further data can be obtained from the corresponding author upon reasonable request.

## Abbreviations

B/M: Breaks per metaphase
FiSH: Fluorescence in situ hybridization
*ATM*_*het*_: *ATM *heterozygotes
DAPI: 4′,6-diamidin-2-phenylindol
